# A Comparative Study on the Efficacy and Safety of Dose Escalation of Luseogliflozin in Type 2 Diabetes Mellitus Patients With Poor Glycemic Control

**DOI:** 10.7759/cureus.35393

**Published:** 2023-02-24

**Authors:** Tadashi Arao, Yosuke Okada, Akira Kurozumi, Yoshiya Tanaka

**Affiliations:** 1 Department of Internal Medicine of Diabetes, Metabolism and Endocrinology and Hematology and Collagen Disease, Japan Labour Health and Safety Organization Kyushu Rosai Hospital, Moji Medical Center, Kitakyushu, JPN; 2 First Department of Internal Medicine, School of Medicine, University of Occupational and Environmental Health, Kitakyushu, JPN

**Keywords:** hemoglobin a1c (hba1c), dose- dependency, sodium-glucose cotransporter 2 (sglt2) inhibitors, luseogliflozin, type 2 diabetes mellitus (t2dm)

## Abstract

Objective

In this study, we aimed to assess the safety and efficacy of the dose escalation of luseogliflozin (LUSEO) in type 2 diabetes mellitus (T2DM) patients with poor glycemic control. To that end, we compared two groups assigned to two different doses of luseogliflozin (LUSEO) for 12 weeks.

Methods

Patients with a hemoglobin A1c (HbA1c) level of 7% or higher already on treatment with luseogliflozin 2.5 mg/day for 12 weeks or longer were randomly assigned to either the 2.5-mg/day group (control group) or the 5-mg/day group (dose-escalation group) of luseogliflozin through the envelope method and were treated for 12 weeks. Blood and urine samples were collected at two different time points: at weeks 0 and 12 after randomization. The primary outcome was the change in HbA1c from the baseline to 12 weeks. The secondary outcomes were changes in the body mass index (BMI), body weight (BW), blood pressure (BP), fasting plasma glucose (FPG), lipid parameters, hepatic function, or renal function from the baseline to 12 weeks.

Results

Based on our findings, HbA1c levels significantly decreased in the dose-escalation group when compared to the control group (p<0.001) at week 12.

Conclusion

For T2DM patients with poor glycemic control under treatment with LUSEO at a dose of 2.5 mg, dose escalation of LUSEO to 5 mg safely improved glycemic control, and this might prove to be an effective and safe treatment option.

## Introduction

Sodium-glucose cotransporter 2 (SGLT2) inhibitors constitute drugs that inhibit SGLT2 in the proximal renal tubule in the kidneys and exert the glucose-lowering effect through urinary glucose excretion. In addition to the glucose-lowering effect, they have been demonstrated to have pleiotropic effects on parameters such as body weight (BW), blood pressure (BP), lipid, visceral fat, or hepatic function [1]. Many reports have also indicated the efficacy of the inhibitors among Japanese people [2-4]. SGLT2 inhibitors that can be prescribed in higher doses in Japan include luseogliflozin, empagliflozin, dapagliflozin, and ipragliflozin. While the overall benefit of canagliflozin, dapagliflozin, and empagliflozin was found to be dose-dependent in a meta-analysis [5], and a retrospective study of empagliflozin showed an increase in dose [6], another meta-analysis [7] has reported that higher doses were as effective as regular doses; hence, it remains unclear whether the effects of SGLT2 inhibitors are dose-dependent. Among SGLT2 inhibitors, luseogliflozin (LUSEO) is a drug used at a minimum dose of 2.5 mg/day, which can be increased to 5 mg/day.

A late phase-III study conducted on 52-week administration of LUSEO in patients in Japan has reported the effects of dose escalation in one arm treated with dose escalation from 2.5 mg/day to 5 mg/day [8]. However, to date, there have been no studies prospectively comparing the effects of LUSEO between doses of 2.5 mg/day and 5 mg/day in real-world clinical settings. In light of this, we conducted this study to investigate the efficacy and safety of dose escalation of LUSEO at 12 weeks after switching the dose to 5 mg/day in type 2 diabetes mellitus (T2DM) patients with poor glycemic control who had been on treatment with LUSEO at a dose of 2.5 mg/day for 12 weeks or longer.

## Materials and methods

Study subjects

The subjects were patients with T2DM who had been treated with oral administration of LUSEO at a dose of 2.5 mg/day for 12 weeks or longer at the outpatient clinic of our department between July 1, 2018, and September 30, 2021, and had poor glycemic control as indicated by a hemoglobin A1c (HbA1c) level of 7% or higher.

Inclusion criteria

Patients who met all of the following criteria were considered to be included in the study: (1) patients aged 20 years or more (regardless of gender), (2) HbA1c of 7.0% or higher, and (3) patients who gave consent to participate in this study.

Exclusion criteria

The exclusion criteria were as follows: (1) patients on insulin, (2) pregnant women or women who were suspected to be pregnant, (3) patients with a history of hypersensitivity to luseogliflozin, (4) patients with serious renal impairment [creatinine clearance (CCr) <30 mL/min; men: creatinine (Cr) >2.4 mg/dL, women: Cr >2.0 mg/dL], and (5) any patients deemed inappropriate for this study by the investigator.

Methods

**Figure 1:**
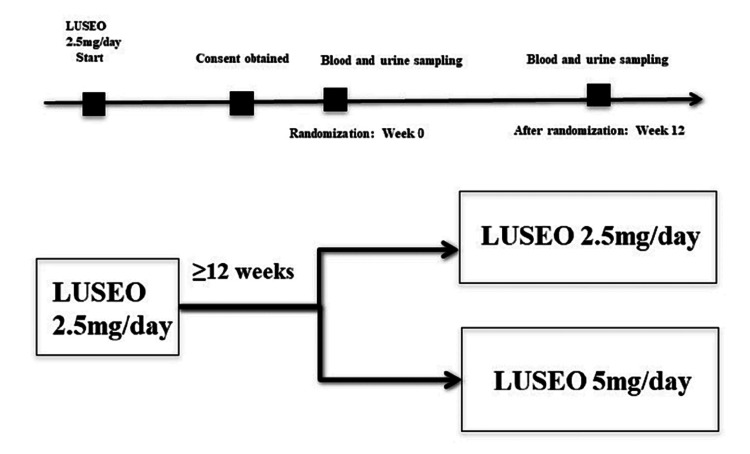
Image depicting the study design

This study was designed as a single-center, randomized, prospective interventional study. The pilot, two-arm randomized controlled trial (RCT) was conducted at the Kyusyu Rosai Hospital Moji Medical Center on June 21, 2018 (approval number: 30-2). The subjects were provided with documents explaining the purpose of the study and they gave informed consent to participate in the study. Afterward, they were randomized into groups using the sealed envelope method (Figure 1).

For assessment, the patients who had been treated with LUSEO at a dose of 2.5 mg/day for 12 weeks or longer and had an HbA1c level of 7% or higher were randomly assigned to either the 2.5-mg/day group (control group) or the 5-mg/day group (dose-escalation group) by using the envelope method and were then treated for 12 weeks. Blood and urine samples were collected at two different time points: at weeks 0 and 12.

During the assessment period, no drugs were added or changed, and no dose escalation was performed. We assessed HbA1c, body mass index (BMI), BW, fasting plasma glucose (FPG), BP, lipid parameters, hepatic function, and renal function.

Outcomes and measurements

The primary outcome was the change in HbA1c from the baseline to 12 weeks. The secondary outcomes were changes in the BMI, BW, systolic blood pressure (SBP) and diastolic blood pressure (DBP), FPG, lipid parameters, hepatic function, and renal function [estimated glomerular filtration rate (eGFR)] from the baseline to 12 weeks.

The lipid parameters measured included triglycerides (TG), high-density lipoprotein cholesterol (HDL-C), and low-density lipoprotein cholesterol (LDL-C). The assessments of hepatic function included measuring aspartate aminotransferase (AST), alanine aminotransferase (ALT), and gamma-glutamyl transferase (γ-GTP).

Statistical analysis

Results are expressed as mean ± standard deviation (SD). Differences in parameters were analyzed by the Mann-Whitney U test, and differences in terms of gender and drugs were analyzed by the chi-square test. A comparison of the two groups was performed using the Wilcoxon signed-rank test. The correlation of changes in HbA1c levels with each parameter was assessed by the Spearman test. The odds ratio (OR) and 95% confidence interval (CI) were used for all data. Statistical analysis was performed using IBM SPSS Statistics 21.0 (IBM Corp., Armonk, NY). A p-value <0.05 was considered statistically significant.

Ethical approval

This study was approved by the Ethics Committee of Kyusyu Rosai Hospital, Moji Medical Center on June 21, 2018 (approval number: 30-2). The subjects were provided with documents explaining the purpose of the study and they gave informed consent to participate in the study.

## Results

The patient characteristics are presented in Table 1.

**Table 1:** Baseline characteristics Differences in parameters were analyzed by the Mann-Whitney U test, and differences in terms of gender and drugs were analyzed by the chi-square test. A p-value <0.05 was considered statistically significant SD: standard deviation; BMI: body mass index; BW: body weight; SBP: systolic blood pressure; DBP: diastolic blood pressure; HbA1c: hemoglobin A1c; FPG: fasting plasma glucose; Cre: creatinine; eGFR: estimated glomerular filtration rate; AST: aspartate aminotransferase; ALT: alanine aminotransferase; γ-GTP: γ-glutamyl transferase; TG: triglycerides; HDL-C: high-density lipoprotein cholesterol; LDL-C: low-density lipoprotein cholesterol; DPP4: dipeptidyl peptidase-4; ARBs: angiotensin II receptor blockers; CCBs: calcium channel blockers

Parameter	Control group	Dose-escalation group	P-value
Sex (male/female)	20 (11/9)	20 (12/8)	0.749
Age, years, mean ± SD	74.9 ± 10.4	70.8 ± 10.8	0.134
Duration, years, mean ± SD	13.7 ± 13.6	11.5 ± 8.3	0.835
BMI, kg/m^2^, mean ± SD	23.4 ± 2.2	24.8 ± 4.4	0.478
BW, kg, mean ± SD	58.9 ± 10.0	62.6 ± 12.9	0.968
SBP, mmHg, mean ± SD	148.9 ± 19.0	151.4 ± 20.7	0.602
DBP, mmHg, mean ± SD	73.8 ± 11.8	83.1 ± 13.3	0.015
HbA1c, %, (NGSP), mean ± SD	7.9 ± 0.8	8.1 ± 0.7	0.369
FPG, mg/dL, mean ± SD	163.9 ± 44.5	163.2 ± 28.8	0.989
Cre, mg/dL, mean ± SD	0.86 ± 0.19	0.75 ± 0.16	0.114
eGFR, mL/min, mean ± SD	59.3 ± 11.1	72.6 ± 17.0	0.007
AST, IU/L, mean ± SD	24.5 ± 11.0	27.2 ± 6.7	0.079
ALT, IU/L, mean ± SD	21.2 ± 9.0	34.9 ± 27.9	0.021
γ-GTP, IU/L, mean ± SD	37.7 ± 25.7	46.2 ± 41.7	0.550
LDL-C, mg/dL, mean ± SD	92.8 ± 46.1	112.6 ± 31.6	0.054
HDL-C, mg/dL, mean ± SD	53.0 ± 14.0	53.8 ± 11.3	0.496
TG, mg/dL, mean ± SD	192.4 ± 250.1	141.2 ± 65.0	0.478
No drug, n (%)	9 (45.0)	8 (40.0)	0.749
Sulfonylurea, n (%)	6 (30.0)	5 (25.0)	0.723
Metformin, n (%)	4 (20.0)	9 (45.0)	0.091
Thiazolidine, n (%)	1 (5.0)	4 (20.0)	0.151
Glinide, n (%)	3 (15.0)	1 (5.0)	0.292
DPP4 inhibitor, n (%)	5 (25.0)	7 (35.0)	0.490
Alpha-glucosidase inhibitor, n (%)	3 (15.0)	1 (5.0)	0.292
Antihypertensive, n (%)	6 (30.0) (ARBs: 6, CCBs: 3)	6 (30.0) (ARBs: 5, CCBs: 2)	1.00
Antilipidemic, n (%)	5 (25.0) (statin: 5, ezetimibe: 0)	7/35.0 (statin: 6, ezetimibe: 1)	0.490

The control and dose-escalation groups included 20 patients (11 men and nine women) and 20 patients (12 men and eight women), respectively, (p=0.749). No significant differences were observed between the two groups in any parameters except for DBP and eGFR.

The patients were advanced in age, with a mean age in the early 70s. The mean BMI was 23.4 ± 2.2 kg/m^2^ in the control group and 24.8 ± 4.4 kg/m^2^ in the dose-escalation group. Both groups included patients who were not very obese. In both groups, 40% of the patients were drug-naïve, and the other most common medications were sulfonylureas, DPP4 inhibitors, and metformin. More than a quarter of the patients were on antihypertensive drugs and drugs for hyperlipidemia.

The control and dose-escalation groups did not differ significantly in terms of other coexisting diseases: hypertension: 30% each; dyslipidemia: 25%, 30%; neuropathy: 55%, 50%; retinopathy: 30%, 20%; diabetic nephropathy: 20%, 15%; cerebral infarction: 5% each; ischemic heart disease: 10%, 5%.

**Figure 2:**
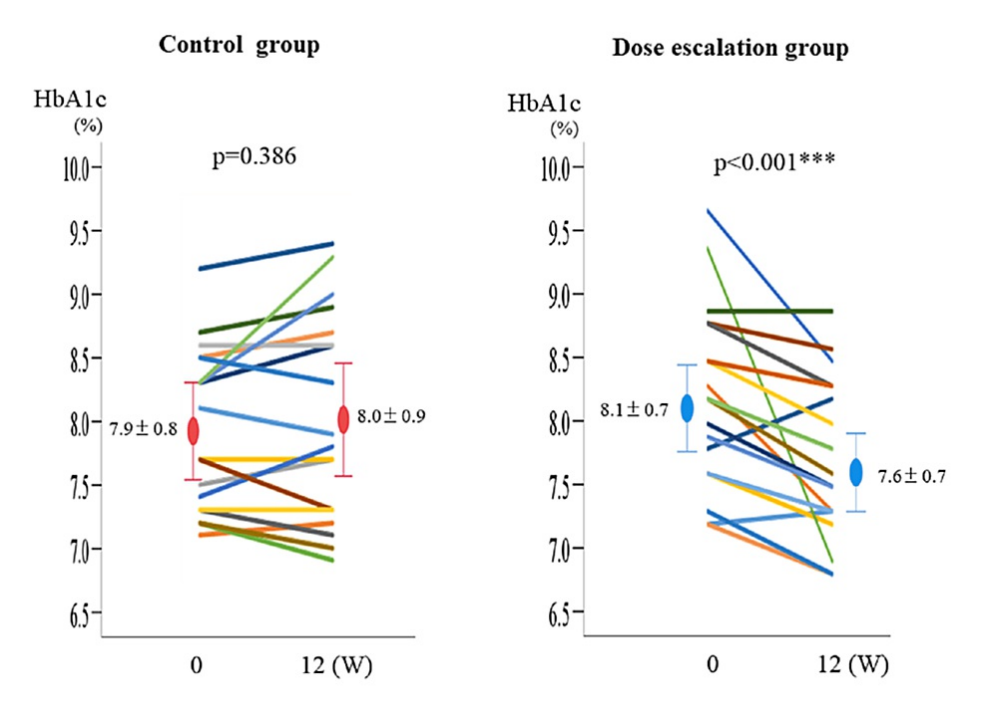
Fluctuations in each case in the control group and the dose-escalation group The comparison of a hemoglobin A1c (HbA1c) between week 0 and week 12 was performed using the Wilcoxon signed-rank test. A p-value <0.05 was considered statistically significant

The fluctuations in each case in the control and dose-escalation groups are shown in Figure 2.

Tables 2a, 2b show changes in each parameter in the control and dose-escalation groups, respectively. Among the parameters, HbA1c levels did not decrease in the control group (7.9 ± 0.8% → 8.0 ± 0.9%) but it significantly decreased in the dose-escalation group (8.1 ± 0.7% → 7.6 ± 0.7%, p<0.001). Table 2c shows the comparison of changes in each parameter at week 12 between the two groups. The HbA1c levels, which were the primary outcome, changed by -0.09 in the control group and -0.51 in the dose-escalation group, showing a significant decrement in the dose-escalation group (p<0.001). Other items (FPG, AST, ALT, LDL-C, HDL-C, DBP) showed a decreasing trend in the dose-escalation group, although there was no significant difference.

**Table 2:** Comparison between parameters within groups at weeks 0 and 12 and across groups at week 12 (a) The comparison of each parameter between week 0 and week 12 in the control group. (b) The comparison of each parameter between week 0 and week 12 in the dose-escalation group. (c) The comparison of changes between the two groups at week 12 The comparison of the two groups was performed using the Wilcoxon signed-rank test. A p-value <0.05 was considered statistically significant SD: standard deviation; BMI: body mass index; BW: body weight; SBP: systolic blood pressure; DBP: diastolic blood pressure; HbA1c: hemoglobin A1c; FPG: fasting plasma glucose; Cre: creatinine; eGFR: estimated glomerular filtration rate; AST: aspartate aminotransferase; ALT: alanine aminotransferase; γ-GTP: γ-glutamyl transferase; TG: triglycerides; HDL-C: high-density lipoprotein cholesterol; LDL-C: low-density lipoprotein cholesterol

(a)			
Control group	Week 0, mean ± SD	Week 12, mean ± SD	P-value
HbA1c, %	7.9 ± 0.8	8.0 ± 0.9	0.386
FPG, mg/dL	163.9 ± 44.5	167.7 ± 41.9	0.723
AST, IU/L	24.5 ± 11.0	25.3 ± 11.7	1.000
ALT, IU/L	21.2 ± 9.0	23.6 ± 10.5	0.038
γ-GTP, IU/L	37.7 ± 25.7	37.2 ± 24.3	1.000
eGFR, mL/min/1.73m^2^	59.3 ± 11.1	59.6 ± 11.8	0.751
LDL-C, mg/dL	92.8 ± 46.1	97.4 ± 46.2	0.395
TG, mg/dL	192.4 ± 250.1	155.9 ± 130.0	0.777
HDL-C, mg/dL	53.0 ± 14.0	54.2 ± 14.3	0.408
BW, kg	58.9 ± 10.0	58.4 ± 10.1	0.020
BMI, kg/m^2^	23.4 ± 2.2	23.1 ± 2.2	0.016
SBP, mmHg	148.9 ± 19.0	141.8 ± 14.0	0.038
DBP, mmHg	73.8 ± 11.8	77.2 ± 11.6	0.379
(b)			
Dose-escalation group	Week 0, mean ± SD	Week 12, mean ± SD	P-value
HbA1c, %	8.1 ± 0.7	7.6 ± 0.7	<0.001
FPG, mg/dL	163.2 ± 28.8	160.5 ± 47.7	0.198
AST, IU/L	27.2 ± 6.7	26.4 ± 7.2	0.491
ALT, IU/L	34.9 ± 27.9	34.5 ± 25.7	0.913
γ-GTP, IU/L	46.2 ± 41.7	45.8 ± 42.3	0.662
eGFR, mL/min/1.73m^2^	72.6 ± 17.0	73.0 ± 21.8	0.350
LDL-C, mg/dL	112.6 ± 31.6	108.9 ± 30.4	0.327
TG, mg/dL	141.2 ± 65.0	157.7 ± 87.1	0.103
HDL-C, mg/dL	53.8 ± 11.3	53.8 ± 13.6	0.856
BW, kg	62.6 ± 12.9	62.2 ± 12.6	0.122
BMI, kg/m^2^	24.8 ± 4.4	24.7 ± 4.3	0.293
SBP, mmHg	151.4 ± 20.7	150.0 ± 18.8	0.872
DBP, mmHg	83.1 ± 13.3	80.5 ± 10.1	0.053
(c)			
	Control group, mean ± SD	Dose-escalation group, mean ± SD	P-value
HbA1c, %	0.1 ± 0.3	-0.5 ± 0.6	<0.001
FPG, mg/dL	3.9 ± 31.3	-2.7 ± 41.5	0.199
AST, IU/L	0.8 ± 5.5	-0.8 ± 6.5	0.652
ALT, IU/L	2.5 ± 4.9	-0.5 ± 10.0	0.321
γ-GTP, IU/L	-0.5 ± 8.9	-0.3 ± 7.7	0.811
eGFR, mL/min/1.73m^2^	0.3 ± 4.3	0.4 ± 8.6	0.379
LDL-C, mg/dL	4.5 ± 18.2	-3.7 ± 13.8	0.122
TG, mg/dL	-36.4 ± 146.5	16.6 ± 50.5	0.286
HDL-C, mg/dL	1.3 ± 6.0	-0.1 ± 6.6	0.683
BW, kg	-0.5 ± 0.9	-0.4 ± 1.2	0.849
BMI, kg/m^2^	-0.2 ± 0.3	-0.1 ± 0.5	0.501
SBP, mmHg	-7.1 ± 15.7	-1.4 ± 22.8	0.144
DBP, mmHg	3.4 ± 12.4	-2.7 ± 12.6	0.316

The changes in HbA1c levels were significantly correlated with baseline HbA1c levels (p=0.02, r=-0.516) (Table 3).

**Table 3:** The correlation of HbA1c level changes with each parameter This correlation was assessed by the Spearman test. A p-value <0.05 was considered statistically significant BMI: body mass index; HbA1c: hemoglobin A1c; FPG: fasting plasma glucose; AST: aspartate aminotransferase; ALT: alanine aminotransferase; eGFR: estimated glomerular filtration rate

Parameter	P	r
Age	0.179	-0.313
Duration	0.448	0.180
BMI	0.997	-0.001
HbA1c	0.020	-0.516
FPG	0.146	0.337
AST	0.258	0.273
ALT	0.654	0.107
eGFR	0.480	0.168

As for safety and tolerability, only one patient in the control group developed an adverse drug reaction (urinary tract infection) during the assessment period, whereas no adverse drug reactions were observed in the dose-escalation group.

## Discussion

To the best of our knowledge, this is the first prospective two-arm intervention study on the effect of dose escalation of SGLT2 inhibitors in the post-marketing period. Absolute changes in HbA1c in three months were significantly decreased in the dose-escalation group (p<0.001) and changes in HbA1c levels were significantly correlated with baseline HbA1c levels. Other parameters (FPG, AST, ALT, LDL-C, HDL-C, and DBP) showed a decreasing trend in the dose-escalation group, although the difference was not statistically significant.

A meta-analysis of 51 randomized clinical trials involving 23,989 patients with T2DM has reported that empagliflozin, dapagliflozin, and canagliflozin significantly decreased regulated glycemia, BW, BP, and HDL in a dose-dependent manner [5]. A retrospective study involving empagliflozin administration six months after dose escalation showed significant decrements in BW, BMI, γ-GTP, TG, FPG, and HbA1c in type 2 diabetic patients [6].

The reason why no significant differences were observed in this study other than in HbA1c levels may be due to the shorter duration of the study compared to other studies. Therefore, more than three months of treatment may be necessary to confirm the various effects of dose increase in regular practice.

In the present study, which involved patients with T2DM who had been receiving LUSEO at a dose of 2.5 mg and had poor glycemic control, dose escalation of LUSEO to 5 mg safely improved glycemic control. Since clinical pharmacology studies showed a dose-dependent increase in urinary glucose excretion from a LUSEO dose of 0.5 mg to 5 mg and a negative correlation between the degree of decrease in blood glucose levels and urinary glucose excretion, the mechanism of the effects of LUSEO appeared to be attributable to an increase in urinary glucose excretion [9]. In the present study, LUSEO was more effective in improving glycemic control with higher HbA1c levels even after dose escalation from 2.5 mg/day to 5 mg/day, although changes in urinary glucose excretion were not examined. This finding appeared to be consistent with that of the aforementioned report.

The changes in HbA1c levels were significantly correlated with baseline HbA1c levels. In a report describing patients who newly started treatment with ipragliflozin at a dose of 50 mg (n=92), the HbA1c levels significantly decreased from 7.6 ± 0.5% at week 0 to 6.9 ± 0.4% at week 12 (p<0.001), and the morning spot urine glucose/creatinine ratio significantly increased from 0.11 to 36.08 (p<0.001), while glycemic control decreased more with higher HbA1c levels and lower urinary glucose excretion at baseline [10].

In addition, Matsumura et al. reported that baseline DBP and TG were independent predictors of HbA1c decrement [6]. In the present study, DBP was also significantly higher in the dose-escalation group at baseline, and differences in patient backgrounds may have contributed to the decrease in HbA1c in the dose-escalation group. On the other hand, the renal function significantly differed at baseline between the two groups in this study: eGFR was 45 mL/min/1.73m^2^ or higher in both groups. In addition, HbA1c change did not correlate with eGFR (p=0.480, r=-0.168). It has been reported that eGFR in this range does not affect the onset of the effects of LUSEO [11]. We assumed that the impact of the differences in renal function on the outcomes was limited.

The present study showed no significant differences in BMI and BW, which were the secondary outcomes. One study has reported that empagliflozin 25 mg compared with 10 mg as an add-on to metformin in patients with type 2 diabetes shows significant weight loss at a BMI of 35 or higher [12]. A possible reason for this was that neither group included any markedly obese patients according to the baseline BMI.

As for safety, a urinary tract infection was detected in one patient in the control group, whereas no adverse drug reactions were observed in the dose-escalation group. A study involving 279 Japanese patients treated with LUSEO has demonstrated the efficacy and safety of treatment with LUSEO at a dose of 2.5 mg for 52 weeks followed by an increased dose of 5 mg for 24 weeks [13]. A systematic review and meta-analysis of RCTs comparing empagliflozin 10 mg versus 25 mg in patients treated for type 2 diabetes and reporting adverse drug reactions as a clinical endpoint reported no difference between doses, consistent with existing reports [14].

Since LUSEO is metabolized through multiple pathways and equally excreted into feces and urine, it has been reported that the pharmacokinetics of LUSEO is unlikely to be affected by the presence or absence or severity of hepatic and renal impairment [15]. However, the sample size was small, and further studies are needed to better evaluate safety between doses. A study in which patients with T2DM started a new antidiabetic therapy (n=235) has reported that adherence to the oral therapy for 12 months was significantly inversely correlated with the most recent HbA1c level [16]. Moreover, the mortality and readmission rates have been reported to be higher in nonadherent patients with diabetes mellitus [17]. Prior studies have reported that medication adherence tends to decrease as the number of medications taken increases [18,19].

The current results suggest that increasing the dose of one tablet of LUSEO from 2.5 mg/day to 5 mg/day may be effective for patients with polypharmacy and insufficient improvement in medication adherence. In addition, 22% of patients with type 2 diabetes have stated that the reason for discontinuing medication was the "cost of medical care," [20] and the cost of increasing the dose of LUSEO from 2.5 mg/day to 5 mg/day is lower than the cost of adding another hypoglycemic agent, making it a viable option from an economic perspective.

This study has a few limitations, which are as follows: (1) The sample size was relatively small: 20 patients in each group. (2) The assessment period was short: only 12 weeks. (3) Insulin secretory ability (serum C-peptide) was not assessed. (4) Fewer adverse drug reactions and other safety parameters observed in this study may not reflect the actual evidence due to the very small sample size. Further studies with larger sample sizes and longer-term follow-ups are needed to validate our findings.

## Conclusions

In patients with T2DM who were receiving LUSEO at a dose of 2.5 mg/day and had poor glycemic control, dose escalation of LUSEO to 5 mg improved glycemic control. The changes in HbA1c levels were significantly correlated with baseline levels. In patients with T2DM with poor glycemic control under treatment with LUSEO at a dose of 2.5 mg, dose escalation of LUSEO to 5 mg safely improved glycemic control, and this might be an effective and safe treatment option.
